# Efficient pH-Responsive Nano-Drug Delivery System Based on Dynamic Boronic Acid/Ester Transformation

**DOI:** 10.3390/molecules28114461

**Published:** 2023-05-31

**Authors:** Weijun Chen, Wanxuan Xie, Guangkuo Zhao, Qi Shuai

**Affiliations:** 1 Department of Child Health Care, Children’s Hospital, Zhejiang University School of Medicine, National Clinical Research Center for Child Health, Hangzhou 310052, China; chenweijun@zju.edu.cn; 2 Collaborative Innovation Center of Yangtze River Delta Region Green Pharmaceuticals, Zhejiang University of Technology, Hangzhou 310014, China; xiewanxuan@zjut.edu.cn (W.X.); zgk7721@163.com (G.Z.)

**Keywords:** pH-responsive, phenylboronic acid, nano-drug, anticancer activity, low cytotoxicity

## Abstract

Chemotherapy is currently one of the most widely used treatments for cancer. However, traditional chemotherapy drugs normally have poor tumor selectivity, leading to insufficient accumulation at the tumor site and high systemic cytotoxicity. To address this issue, we designed and prepared a boronic acid/ester-based pH-responsive nano-drug delivery system that targets the acidic microenvironment of tumors. We synthesized hydrophobic polyesters with multiple pendent phenylboronic acid groups (PBA-PAL) and hydrophilic PEGs terminated with dopamine (mPEG-DA). These two types of polymers formed amphiphilic structures through phenylboronic ester linkages, which self-assembled to form stable PTX-loaded nanoparticles (PTX/PBA NPs) using the nanoprecipitation method. The resulting PTX/PBA NPs demonstrated excellent drug encapsulation efficiency and pH-triggered drug-release capacity. In vitro and in vivo evaluations of the anticancer activity of PTX/PBA NPs showed that they improved the pharmacokinetics of drugs and exhibited high anticancer activity while with low systemic toxicity. This novel phenylboronic acid/ester-based pH-responsive nano-drug delivery system can enhance the therapeutic effect of anticancer drugs and may have high potential for clinical transformations.

## 1. Introduction

The rapid development of nanotechnology and the emergence of intelligent precision medicine have made nano-drug delivery systems (NDDS) possess broad applications in the diagnosis and treatment of cancer [1,2]. The advances in recent research have demonstrated that NDDS could significantly improve the efficiency of chemotherapy drugs and overcome many of their inherent defects. Nanoparticle formulations can improve the solubility and stability of drugs and thus prolong their blood circulation times [3,4]. Secondly, targeting delivery of antitumor drugs via NDDS can enhance drug accumulation at tumor sites, improve their pharmacokinetic parameters and bioavailability, and reduce systemic toxicity [5,6]. In addition, these above-mentioned and other features of NDDS can be further improved through rational design and modification, making it possible to address many critical challenges facing drug delivery and cancer therapy.

Over recent decades, stimuli-responsive nano-drug delivery systems have been widely used in the field of biomedicine, including triggered drug delivery vehicles, biosensors, and supramolecular assemblies [7,8,9,10]. Intelligent nano-drug delivery systems that are especially responsive towards specific tumor microenvironment have always been one of the research hotspots. By designing and preparing nano-formulations capable of identifying and responding to tumor-specific microenvironments, such as lower pH [11,12], higher redox potential [13,14], and higher concentrations and activities of specific enzymes [15], therapeutic drug can be specifically released at the tumor sites in a dynamically controllable manner. It has been well demonstrated that tumor tissues normally have an acidic microenvironment (pH 6.2–6.9) caused in part by lactic acid accumulation and high glycolysis rate in rapidly growing tumor cells [16]. Therefore, a lot of novel tumor pH-responsive nano-drug delivery systems have been developed, in which the controlled release of drugs was triggered by the weak acidic microenvironment in tumor tissues or cells [17,18,19,20,21,22]. These pH-responsive nano-drug delivery systems are generally stable under the physiological pH conditions (pH 7.4). When transferred to tumor sites with lower pH, they undergo rapid dissolution, achieving selective release of loaded drugs to the tumor cells [18]. There are normally two strategies to prepare nano-drug delivery systems: (1) loading drug into vehicles via pH-sensitive covalent or non-covalent bonds [23,24,25] or (2) altering or destroying the vehicle structures through the cleavage of pH-sensitive chemical bonds or the protonation of basic groups. A variety of pH-sensitive functional groups or chemical bonds have been successfully applied to the design and development of pH-responsive nano drug delivery systems, such as acetal [26], ortho-ester [27], hydrazone [28], imine [29], vinyl ether [30], *cis*-butenediamide [31], and β-thiopropionate [32].

Recently, pH-responsive nano-drug delivery systems based on dynamic boronic acid/ester transformation have received considerable attentions [33,34,35]. As a multifunctional chemical group, boronic acid (BA) has been widely used in the field of biomedicine, including biosensors and probes, chromatographic separation of sugar, RNA affinity columns, and drug delivery systems [36,37,38]. In an aqueous solution with pH higher than the pKa of BA, the formation of boronic ester (BE) is promoted by strong binding affinity of BA to diols (polyhydroxy compounds containing 1,2- or 1,3-diols and catechols) [39,40]. This process is reversible, and the BE structure would dissociate when the pH of medium becomes lower than the pKa of BA [41]. This unique property of BA is of great significance for the development of pH-responsive nanodrugs. At the same time, since diols are widely naturally occurring, this dynamic boronic acid/ester bond might have better biocompatibility than other pH-responsive structures, such as imines which are readily hydrolyzed to ketones and amines.

At present, there are two main strategies for the development of pH-responsive nano-drug delivery systems based on boronic acid/ester transformation. For the first one, boronic esters are introduced into vehicles through condensation reactions between BA and diols. Chen et al. [42] synthesized amphiphilic block copolymer dextran-*block*-polylactide (Dex-*b*-PLA), in which the hydrophilic dextran chain was partially modified with 3-carboxy-5-nitrophenylboronic (CNPBA). Under physiological conditions (pH 7.4), the resulting copolymers could self-assemble into pH-responsive nanoparticles with shell crosslinked by CNPBA. For the other one, drugs are covalently connected to the vehicles via boronic ester linkages. For example, the Messersimth [40] and Yang [43] groups have successfully prepared pH-responsive nanodrugs, which used Bortezomib (BTZ) bearing a phenylboronic acid (PBA) structure as an active drug and amphiphilic block copolymers modified with catechol as delivery vehicles. Furthermore, many other drugs with diol or catechol moieties have also been explored to be combined with PBA-modified vehicles and to construct pH-responsive nanodrugs, such as Capecitabine (CAPE) [44], Andrographolide (AND) [45], glycoprotein [46], and curcumin (unsaturated 1,3-diketone are also applicable) [47].

However, many challenges still need to be addressed for the clinical applications of these pH-responsive nanodrugs based on dynamic boronic acid/ester transformations. First of all, the low pKa of boronic acid and its derivatives lead to poor responsiveness to the weakly acidic microenvironment of tumors [48]. In addition, the hydrophilicity of boronic acid may influence the hydrophile-lipohile balance value of amphiphilic block copolymers and thus affect the load efficiency of drugs [42]. Herein, we described a novel phenylboronic acid/ester-based pH-responsive nano-drug delivery system, which achieved efficient delivery of the anticancer drug Paclitaxel (PTX) to cancer cells. Hydrophobic polyesters bearing multiple pendent phenylboronic acid groups were synthesized via ring-opening polymerization (ROP) and click chemistry. Catechol-modified hydrophilic polymers were prepared by attaching dopamine (DA) to carboxyl terminated mPEG. It is found that, under neutral conditions, these two kinds of polymers formed amphiphilic structures via phenylboronic ester linkages, which then self-assembled to form stable PTX-loaded nanoparticles (PTX/PBA NPs) by nanoprecipitation method. The particle sizes, PDI, Zeta potential, morphology, and encapsulation efficiency of resulting NPs were characterized by DLS, TEM, and HPLC. The in vitro drug release assays showed that PTX/PBA NPs had good pH-responsiveness and excellent pH-triggered drug release capacity. Moreover, the in vitro and in vivo evaluations of anticancer activity showed that PTX/PBA NPs could effectively improve the pharmacokinetics of drugs and exhibited high anticancer activity and low systemic toxicity.

## 2. Results

### 2.1. Synthesis and Characterization of Polymers

**Figure 1:**
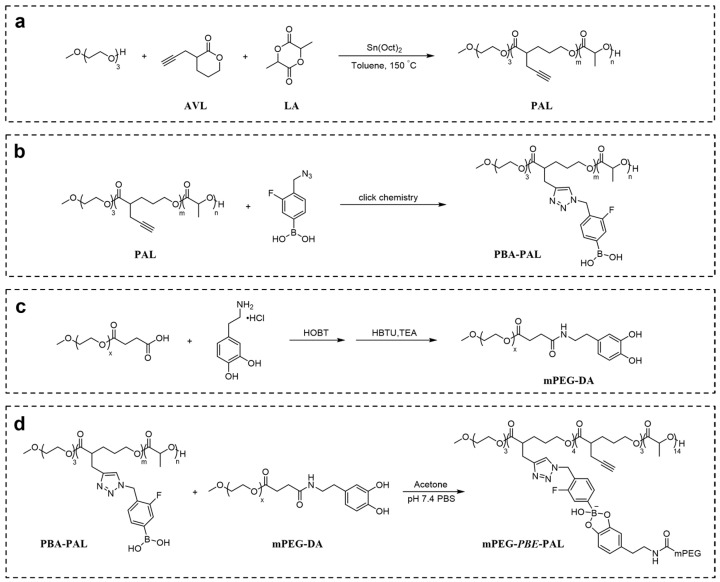
Construction of pH-responsive polymeric nano-carrier based on dynamic boronic acid/ester transformation. Synthesis of (**a**) PAL; (**b**) PBA-PAL; (**c**) mPEG-DA; (**d**) mPEG-*PBE*-PAL.

**Figure 2:**
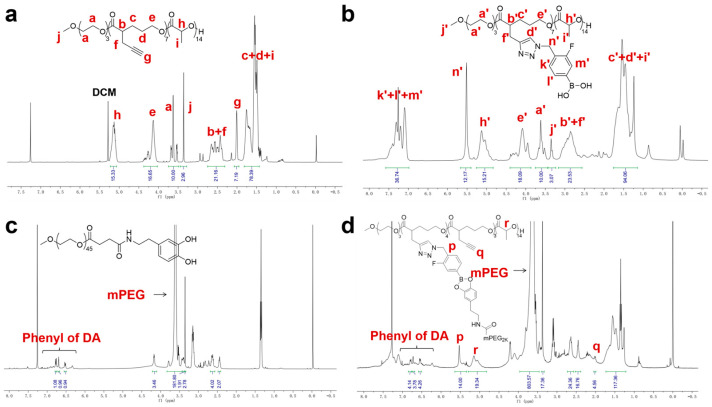
The representative ^1^H NMR of polymers. (**a**) PAL_2K_; (**b**) PBA-PAL_2K_; (**c**) mPEG_2K_-DA; and (**d**) mPEG-*PBE*-PAL.

To construct a pH-responsive nano-drug delivery system based on dynamic boronic acid/ester transformation, we designed and synthesized hydrophobic polymers with multiple pendent phenylboronic acid groups (PBA-PAL) and dopamine (with catechol structure)-terminated hydrophilic polymers (mPEG-DA), respectively (Figure 1). Firstly, hydrophobic polyesters PAL_2K_ and PAL_4K_ bearing alkynyl groups were prepared via ring-opening polymerization (ROP). Additionally, then, multiple 3-fluorophenyl boronic acid (with low pKa) molecules were efficiently attached to PAL via copper-catalyzed azide-alkyne cycloaddition (CuAAC), affording phenylboronic acid-functionalized hydrophobic copolymers PBA-PAL with high grafting rates. The structure of PBA-PAL was confirmed by ^1^H NMR spectra which indicated the presence of the methylene peaks of fluorophenyl boronic acid (5.52 ppm) and the characteristic peaks of 1,2,3-triazole (7.58–6.98 ppm) (Figure 2b). Dopamine-terminated hydrophilic polymers mPEG_2K_-DA and mPEG_4K_-DA were synthesized via amidation of carboxylated mPEG with dopamine hydrochloride (Figure 1c) and characterized by ^1^H NMR spectra (Figure 2c). The phenyl peaks of dopamine (6.76–6.53 ppm) and the characteristic peaks of mPEG (3.82–3.46 ppm) were clearly observed. GPC data showed that these polymers had well-controlled molecular weights and narrow distributions (PDI < 1.4) (Table 1).

We next evaluated the binding ability of PBA-PAL and mPEG-DA in a weak alkaline environment (pH = 7.4). The formation of amphiphilic copolymers with boronic ester linkages was characterized by ^1^H NMR. The simultaneous appearance of characteristic peaks of hydrophobic PBA-PAL and hydrophilic mPEG-DA indicated the effective binding of them, affording amphiphilic copolymers mPEG-*PBE*-PAL. However, it was found that mPEG-DA was not completely connected to PBA-PAL. The presence of the alkynyl peaks of PBA-PAL (2.00 ppm) showed that the binding efficiency of these two types of polymers is about 40–50% (Figure 2d).

### 2.2. Preparation and Characterization of PTX-Loaded Nanoparticles

We examined the self-assembly of hydrophobic copolymer PBA-PAL and hydrophilic mPEG-DA using a nanoprecipitation method, expecting to obtain pH-responsive PTX-loaded PBA-PAL NPs (PTX/PBA NPs). As control, non-pH responsive PTX/mPEG-PAL NPs were also prepared by self-assembly of amphiphilic graft copolymer mPEG-*g*-PAL. At the beginning, PBA-PAL_2K_ and mPEG_2K_-DA were selected as the model polymers to optimize formulation conditions. A serial of PTX/PBA NPs with different feed molar ratios of polymers was prepared and characterized by DLS and HPLC. As revealed in Table 2, all NPs presented applicable sizes and low PDI. In addition, the encapsulation efficiency (EE) of NPs increased with the feed ratio of mPEG-DA from 0.5 to 7. A highest EE of 96.1% was obtained when the molar feed ratio of mPEG-DA to PBA-PAL_2K_ reached up to 7:1. However, the highest drug loading (DL) of 2.0% was detected only when the molar feed ratio of mPEG-DA to PBA-PAL_2K_ was set at 1:1. Further optimization for the preparation of PTX-loaded NPs was conducted by using PBA-PAL and mPEG-DA with different molecular weights and the same molar feed ratio of 1:7, the results of which were summarized in Table 3. It was still the combination of PBA-PAL_2K_ mPEG_2K_-DA that afforded the highest EE and the highest DL. Additionally, thus, the final formulation was determined as [PBA-PAL_2K_]:[mPEG_2K_-DA] = 1:7. As comparison, we also prepared and characterized non-pH responsive PTX/mPEG-PAL NPs with amphiphilic mPEG-*g*-PAL (**1-3**) that were synthesized by following our previous procedures (Figure S9–S11) [49]. No formation of NPs was detected for mPEG-*g*-PAL **3** in which 7 mPEG_2k_ chains were grafted to the backbone of PAL_2K_ (Table S1). It might be explained that the large proportion of hydrophilic segments in mPEG-*g*-PAL **3** led to the loss of its self-assembly ability. For mPEG-*g*-PAL **1** with only one mPEG_2K_ chain on PAL backbone, it could efficiently form nanoparticles with applicable sizes and low PDI, as well as reasonable EE and DL.

### 2.3. Morphology, Stability, and pH-Responsiveness of PTX-Loaded Nanoparticles

**Figure 3:**
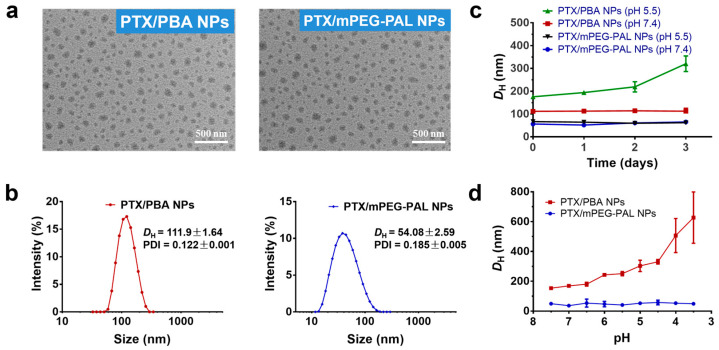
Characterization of PTX NPs. (**a**) TEM images of PTX/PBA NPs and PTX/mPEG-PAL NPs; (**b**) DLS of PTX/PBA NPs and PTX/mPEG-PAL NPs; (**c**) Variation of *D*_H_ of NPs in PBS (pH = 7.4) and (pH = 5.5); (**d**) Evaluation of pH responsiveness of NPs.

The morphology of these PTX-loaded NPs prepared under optimized conditions was studied by transmission electron microscopy (TEM) and dynamic light scattering (DLS). As shown in Figure 3a,b, all NPs presented uniform spherical structures, applicable particle sizes, and low PDI. High stability and high sensitivity to pH changes of NPs are of great importance for efficient and targeting delivery of antitumor drugs. In this study, the stability and pH-responsiveness of PTX-loaded NPs were investigated by DLS. Noticeably, as showed in Figure 3c, PTX/PBA NPs exhibited great stability in pH 7.4 while increased particle sizes were observed in pH 5.5 medium over 3 days, which illustrated the PTX/PBA NPs collapsed gradually with the cleavage of boronic ester in acidic environment. As expected, the sizes of PTX/mPEG-PAL NPs did not change both at pH 7.4 and pH 5.5 over 3 days. We further evaluated the pH responsiveness of these NPs by monitoring the size of PTX/PBA NPs and PTX/mPEG-PAL NPs at different pH values. As shown in Figure 3d, the size of PTX/PBA NPs increased and became inhomogeneous with the decrease of pH, while that of PTX/mPEG-PAL NPs remained relatively stable, which demonstrated that PTX/PBA NPs showed good stability and excellent pH-sensitivity.

### 2.4. In Vitro PTX Release Profiles

**Figure 4:**
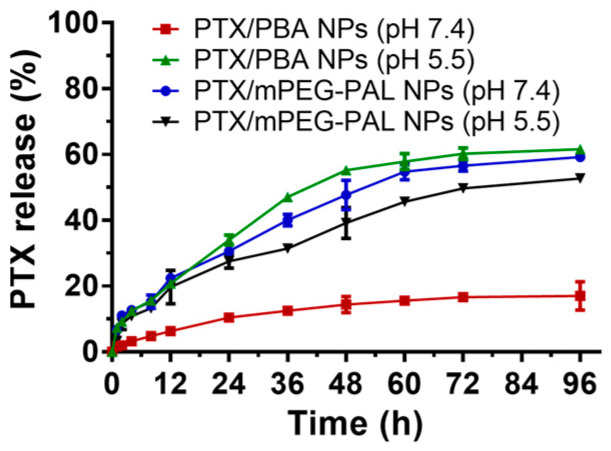
In vitro drug release profile of PTX nanoparticles.

To test and verify the pH-responsive drug release properties of PTX/PBA NPs, we next investigated the release profiles of PTX from these NPs in PBS with different pH values (pH 7.4 and pH 5.5), and the PTX/mPEG-PAL NPs were used as the control group. As shown in Figure 4, only ~20% of the PTX was released from the PTX/PBA NPs at pH 7.4 while more than 60% at pH 5.5 within 96 h, which confirmed that PTX was slowly released from the PTX/PBA NPs at pH 7.4 and a significantly accelerated release profile was observed at pH 5.5, whereas rapid release of PTX from PTX/mPEG-PAL NPs was detected for both at pH 7.4 and pH 5.5, which might contribute to the weak interaction between PTX and polymeric vehicles [50]. These results suggested that the PTX/PBA NPs could be a promising pH-responsive nano-drug delivery system.

### 2.5. Assessment of In Vitro Cytotoxicity and Cellular Uptake

**Figure 5:**
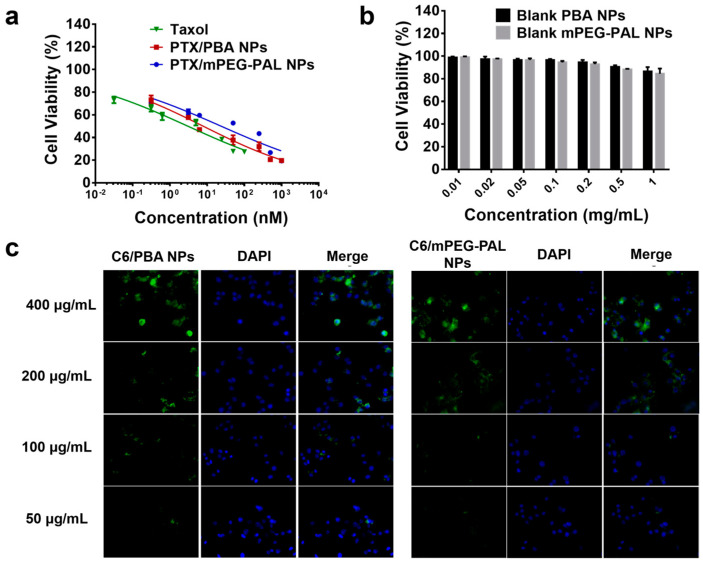
Cytotoxicity and cellular uptake evaluation. (**a**) Cell viability of DU145 cells after incubated with PTX NPs for 24 h; (**b**) Cell viability of DU145 cells after incubated with blank NPs for 24 h; (**c**) Analysis of the cellular uptake of NPs in DU145 cells, scale bar = 75 μm.

Subsequently, we assessed the in vitro cytotoxicity of PTX-loaded NPs by MTT (3-(4,5-dimethylthiazol-2-yl)-2,5-diphenyltetrazolium bromide) assays. Free Taxol and PTX/mPEG-PAL NPs were used as the control group. As shown in Figure 5a, the half-maximal inhibitory concentrations (IC_50_) of Taxol, PTX/PBA NPs, and PTX/mPEG-PAL NPs were 3.04, 7.86, and 23.8 nM, respectively. The cytotoxicity of PTX/PBA NPs and PTX/mPEG-PAL NPs was slightly lower than that of free Taxol but still maintained at an effective level. The reduced cytotoxicity could be explained by the extended release of therapeutically active PTX from nanoparticle formulations, which was consistent with previous work [51]. Moreover, we also investigated the cytotoxicity of drug free PBA NPs and mPEG-PAL NPs, and the results showed that all these NPs exhibited low cytotoxicity, indicating the low cytotoxicity of these synthesized polymeric vehicles (Figure 5b). To further evaluate the cellular uptake of NPs, we prepared fluorescent dye coumarin-6 (C6)-loaded C6/PBA NPs and C6/mPEG-PAL NPs which were observed by fluorescence microscope. Due to the similar formulations, C6/PBA NPs and C6/mPEG-PAL NPs exhibited consistent cellular uptake efficiency, both of which were enhanced with the increase of the nanoparticle concentrations (Figure 5c).

### 2.6. Pharmacokinetics Studies

**Figure 6:**
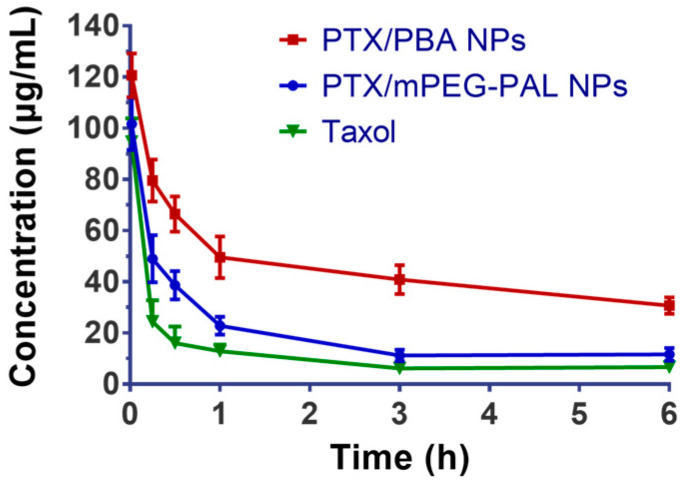
In vivo drug plasma concentration–time profiles in ICR mice.

We then studied the in vivo pharmacokinetics of the NPs after intravenous administration of free Taxol, PTX/PBA NPs, and PTX/mPEG-PAL NPs in order to investigate whether the NPs could prolong the blood circulation time of loaded drugs. As shown in Figure 6 and Table 4, both PTX/PBA NPs and PTX/mPEG-PAL NPs presented obvious longer half-lives, while rapid clearance of Taxol was observed. In addition, the area under the concentration time curve (AUC (0–t)) of PTX/PBA NPs is 4.0- and 2.4-fold that of Taxol and PTX/mPEG-PAL NPs, respectively. Compared with PTX/PBA NPs, the poorer pharmacokinetic parameters of PTX/mPEG-PAL NPs might be attributed to the instability of the formulation of NPs. The above results indicated that the PTX/PBA NPs could improve the pharmacokinetics of PTX significantly through reasonable design and preparation.

### 2.7. In Vivo Antitumor Activity

**Figure 7:**
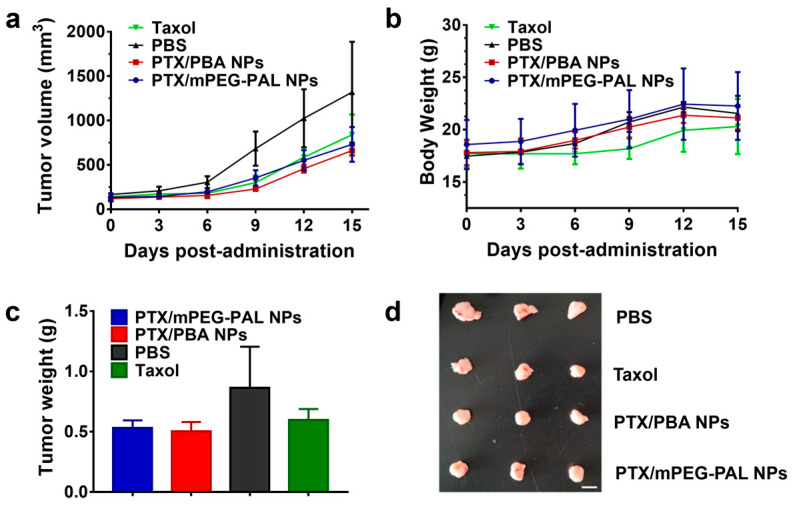
In vivo antitumor activity studies. (**a**) Changes in tumor volumes; (**b**) Body weight changes in mice; (**c**) Average weight of tumors at the end of treatment; (**d**) Ex vivo tumor images at the end of treatment.

The excellent in vitro antitumor activity of PTX/PBA NPs promoted us to further verify their therapeutic efficacy in a tumor xenograft-bearing mouse model. Firstly, we established the human DU145 tumor model in BALB/c nude mice. After intravenous injection of PBS, free Taxol, PTX/PBA NPs and PTX/mPEG-PAL NPs through the tail vein of mice on day 0, 3 and 6, respectively, the tumor volume and mice weight in each group were recorded. As shown in Figure 7a, the growth of tumor was suppressed by the administration of Taxol and PTX-loaded NPs while the tumor in PBS-treated mice grew rapidly. It is worth noting that the tumor treated with Taxol and PTX-loaded NPs was completely inhibited in the first 6 days. Additionally, after that, the tumor started to grow slowly at varying degrees, which might be contributed to the systemic metabolism of drugs. Moreover, the mice showed an uptrend in body weight after the administration of PBS and PTX-loaded NPs. The body weight of Taxol-treated mice remained almost unchanged in the first 9 days and then showed a slow uptrend (Figure 7b). No loss of weight was observed for all mice in the test. The photograph and weight data of the tumor collected from mice at the end of treatment were summarized in Figure 7c,d. Taxol, PTX/PBA NPs, and PTX/mPEG-PAL NPs showed a better therapeutic efficacy than PBS. However, PTX-loaded NPs did not exhibit superiority of antitumor activity over Taxol, which might have contributed to the low dose of these PTX-loaded NPs.

**Figure 8:**
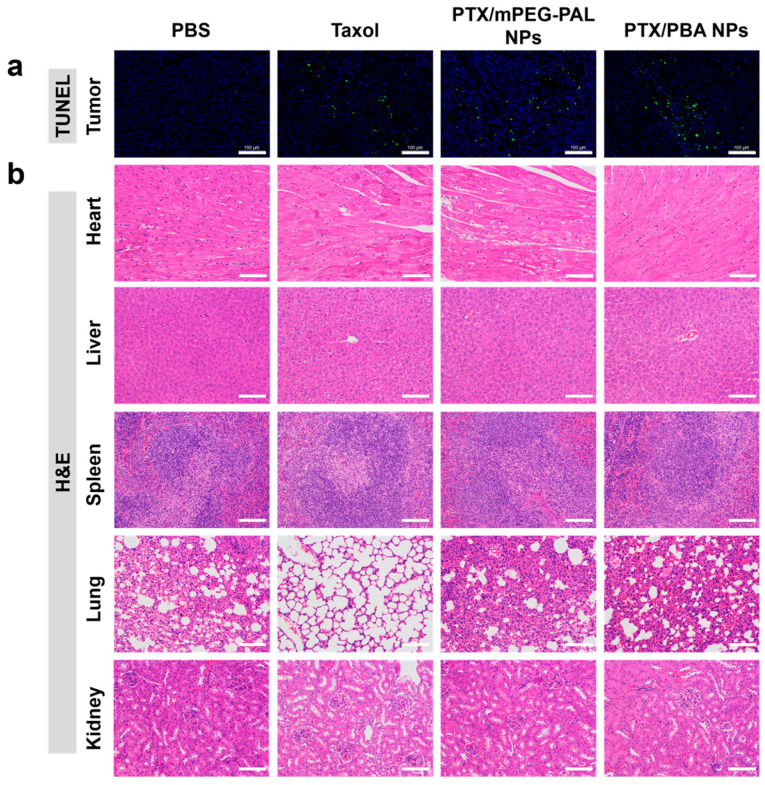
(**a**) TUNEL assay of the tumors and (**b**) H&E staining of the major organs excised. Scale bar = 100 μm.

At last, histological analyses of tumor and major organs were carried out by the terminal deoxynucleotidyl transferase-mediated dUTP nick-end labeling (TUNEL) assay and hematoxylin and eosin (H&E) staining. In the TUNEL assay shown in Figure 8a, compared with the mice receiving PBS treatments, the administration of PTX/PBA NPs, PTX/mPEG-PAL NPs, and Taxol caused a dramatic increase of the cell apoptosis rate in tumors. All PTX-loaded NPs showed similar antitumor activities to free Taxol. In addition, no necrosis or cell death was observed from the results of H&E staining of major organs, indicating the in vivo safety of both PTX/PBA NPs and PTX/mPEG-PAL NPs (Figure 8b).

## 3. Materials and Methods

### 3.1. Materials

Triethylene glycol monomethyl ether (98%) and mPEGs (M_n_ = 2000, 4000) were purchased from TCI chemicals (Shanghai, China). Lithium diisopropylamide (2 M sol. in THF/n-heptane/ethylbenzene), N,N,N’,N’’,N’’-pentamethyldiethylenetriamine (99%), and cuprous bromide (99%) were purchased from Aladdin chemicals (Shanghai, China). Propargyl bromide (98%), hexamethylphosphoramide (HMPA) (98%), δ-valerolactone (98%), Sn(Oct)_2_ (97%), D,L-lactide (99%), succinic anhydride (99%), dopamine hydrochloride (98%), triethylamine (99.5%), DMAP (98%), 3-fluoro-4-methylphenylboronic acid (98%), N-bromosuccinimide (98%), AIBN (98%), HOBT (99%), HBTU (99%), and PTX were purchased from Energy chemicals (Shanghai, China). All solvents were dried before use.

### 3.2. Instruments

^1^H NMR spectra were recorded at 400 MHz (Bruker AVVANCE DRX-400 NMR spectrometer, Bruker, Switzerland). CDCl_3_ and DMSO-d_6_ were used as deuterated solvents. Chemical shifts (δ, ppm) were determined with internal solvent signal as reference (CHCl_3_: δ = 7.26, DMSO-d_6_: δ = 2.50). Gel permeation chromatography (GPC) (Waters 1515-2414, Waters, Milford, MA, USA) was used to determine the number- and weight-average molecular weights (M_n_ and M_w_) and polydispersity (M_w_/M_n_) of copolymers in THF at 30 °C with 1 mL/min flow rate. Polystyrene standards were used to calibrate the instrument. Transmission electron microscopy (TEM) images were taken with a Hitachi HT7700 instrument operating at an accelerating voltage of 120 KV. The particle size and polydispersity index (PDI) of polymeric nanoparticles were determined by dynamic light scattering (DLS) equipped with a Malvern Nano ZS90 Zetasizer, a 632.8 nm He-Ne laser, and a 173° backscatter detector.

### 3.3. General Procedures for the Synthesis of Poly (AVL-co-LA) (PAL)

All glass apparatuses for the reaction were flame-dried before use. To a round-bottom flask purged with N_2_ was added a solution of triethylene glycol monomethyl ether (0.24 g, 1.5 mmol), D, L-Lactide (1.50 g, 10.4 mmol), and AVL (1.50 g, 10.9 mmol) in anhydrous toluene (25 mL), followed by stirring at 150 °C for 10 min and the addition of 6–7 drops of Sn(Oct)_2_. The stirring continued for another 48 h. After that, the mixture was cooled down to room temperature and diluted with dichloromethane (DCM, 200 mL), followed by addition of a few drops of 0.1 M HCl. The organic layer was washed by water until the aqueous layer turned neutral. The residue was purified by precipitation of DCM:hexane (1:10) three times to afford the poly(AVL-co-LA)_2K_ (AVL:LA = 1:1) (PAL_2K_) (75% yield). ^1^H NMR (400 MHz, CDCl_3_, δ): 5.24–4.99 (m, 14H), 4.36–4.23 (m, 3H), 4.18–4.04 (m, 14H), 3.75–3.48 (m, 10H), 3.36 (s, 3H), 2.75–2.31 (m, 21H), 2.00 (s, 7H), 1.82–1.43 (m, 70H).

Poly(AVL-co-LA)_4K_ (AVL:LA = 1:1) (PAL_4K_) was synthesized similarly with different loading of monomers (yield 81%). ^1^H NMR (400 MHz, CDCl_3_, δ): 5.24–4.99 (m, 28H), 4.36–4.23 (m, 3H), 4.18–4.04 (m, 28H), 3.75–3.48 (m, 10H), 3.36 (s, 3H), 2.75–2.31 (m, 42H), 2.00 (s, 14H), 1.82–1.43 (m, 140H) (Figure S2).

### 3.4. Synthesis of PBA-PAL Polymer

PAL_2K_ or PAL_4K_ (1 equiv. in terms of alkynyl group), 4-azidomethyl-3-fluorophenylboronic acid (3 equiv.), cuprous bromide (1 equiv.), and pentamethyldiethylenetriamine (1 equiv.) were added in anhydrous DMF under N_2_. The mixture was stirred in the dark at 50 °C for 24 h. After the reaction, the mixture was dialyzed in water for 2 days (molecular weight cutoff of 2 kD), and vacuum freeze-drying afforded PBA-PAL_2K_ and PBA-PAL_4K_ (75–92%).

PBA-PAL_2K_: ^1^H NMR (400 MHz, CDCl_3_, δ): 7.58–6.98 (m, 28H), 5.52 (s, 12H), 5.26–4.83 (m, 14H), 4.40–3.87 (m, 17H), 3.76–3.44 (m, 10H), 3.35 (s, 3H), 3.17–2.56 (m, 21H), 1.75–1.15 (m, 70H).

PBA-PAL_4K_: ^1^H NMR (400 MHz, CDCl_3_, δ): 7.58–6.98 (m, 56H), 5.52 (s, 24H), 5.26–4.83 (m, 28H), 4.40–3.87 (m, 34H), 3.76–3.44 (m, 10H), 3.35 (s, 3H), 3.17–2.56 (m, 42H), 1.75–1.15 (m, 140H) (Figure S5).

### 3.5. General Procedures for the Synthesis of mPEG-DA

mPEG-COOH was synthesized in our laboratory by following reported procedures [51]. Take the synthesis of mPEG_2K_-DA for example. A solution of mPEG_2K_-COOH (2.0 g, 1 mmol), dopamine hydrochloride (379.28 mg, 2 mmol), and 1-Hydroxybenzotriazole (HOBT, 472.52 mg, 3.5 mmol) in anhydrous DCM was stirred under N_2_ and at 25 °C for 15 min. Additionally, then benzotriazole-N,N,N′,N′-tetramethylurea hexafluorophosphate (HBTU, 60 mg, 2 mmol) and triethylamine (486.4 μL, 3.5 mmol) were added to the mixture, and the stirring continued for another 3 h. After the reaction, the mixture was diluted with DCM (50 mL) and washed with 1 M HCl. The aqueous phase was extracted with DCM three times (3 × 50 mL). Then, the combined organic layer was dried over anhydrous Na_2_SO_4_, filtered, and concentrated under vacuum. The residue was purified by precipitation of DCM:diethyl ether (1:10) three times to afford the mPEG_2K_-DA (1.98 g, 92%). ^1^H NMR (400 MHz, CDCl_3_, δ): 6.76 (d, 1H), 6.70 (d, 1H), 6.53 (d, 1H), 3.82–3.46 (m, 181H), 3.44–3.40 (t, 2H), 3.36 (s, 3H), 2.73–2.55 (m, 4H), 2.43 (m, 3H).

mPEG_4K_-DA was synthesized in a similar way (yield 88%). ^1^H NMR (400 MHz, CDCl_3_, δ): 6.76 (d, 1H), 6.70 (d, 1H), 6.53 (d, 1H), 3.82–3.46 (m, 363H), 3.44–3.40 (t, 2H), 3.36 (s, 3H), 2.73–2.55 (m, 4H), 2.43 (m, 3H) (Figure S6).

### 3.6. Binding Affinity of PBA-PAL and mPEG-DA

A solution of PBA-PAL (1 equiv.) and mPEG-DA (7 equiv.) in acetone was added dropwise into PBS (pH 7.4) with stirring. After removal of the organic solvent, the mixture was dialyzed in PBS (pH 7.4). Vacuum freeze-drying afforded binding polymers which were characterized by ^1^H NMR.

### 3.7. Preparation and Characterization of PTX/PBA NPs and PTX/mPEG-PAL NPs

PTX-loaded NPs including pH-responsive PTX/PBA NPs, and control non-pH responsive PTX/mPEG-PAL NPs were prepared by a nanoprecipitation method described in detail as follows.

PTX/PBA NPs: Drug PTX (1 mg) and polymers PBA-PAL and mPEG-DA with different ratios were dissolved in acetone (2 mL). The mixture was stirred for 24 h and added dropwise into deionized water (10 mL) with stirring. After the addition, stirring continued for another 6 h, and then the acetone was removed using a rotary evaporator under reduced pressure, which yielded an aqueous solution of PTX/PBA NPs at a concentration of 0.1 mg/mL.

PTX/mPEG-PAL NPs: A solution of mPEG-g-PAL in acetone (2 mL) was added dropwise into deionized water (10 mL) with stirring. After the addition, stirring continued for another 30 h, and then acetone was removed using a rotary evaporator under reduced pressure, which yielded an aqueous solution of PTX/mPEG-PAL NPs at a concentration of 0.1 mg/mL.

The particle size and polydispersity index (PDI) of the PTX-formulated NPs were measured by dynamic light scattering (DLS) using a particle size analyzer (Zetasizer, Malvern, UK).

The drug loading (DL%) and encapsulation efficiency (EE%) of the PTX-loaded NPs were calculated by the following equations:

(1) $$\mathrm{DL}\;\left(\%\right)=\frac{\mathrm{Weight}\;\mathrm{of}\;\mathrm{the}\;\mathrm{drug}\;\mathrm{in}\;\mathrm{NPs}}{\mathrm{Weight}\;\mathrm{of}\;\mathrm{the}\;\mathrm{polymer}\;\mathrm{and}\;\mathrm{initial}\;\mathrm{drug}}\times 100\%$$

(2) $$\mathrm{EE}\;\left(\%\right)=\frac{\mathrm{Weight}\;\mathrm{of}\;\mathrm{the}\;\mathrm{drug}\;\mathrm{in}\;\mathrm{NPs}}{\mathrm{Weight}\;\mathrm{of}\;\mathrm{the}\;\mathrm{initial}\;\mathrm{drug}}\times 100\%$$

### 3.8. In Vitro Drug Release Assay

The in vitro release profiles of PTX from the corresponding NPs were investigated using a dialysis method. Briefly, 4 mL of PTX-loaded NPs (PTX/PBA NPs and PTX/mPEG-PAL NPs) solutions with 0.1 mg/mL PTX equivalent concentration was prepared. A total of 1 mL of the resulting solution was taken to quantify its initial concentration, and the remaining 3 mL was loaded into a dialysis bag (molecular weight cutoff of 4 kD) which was then submerged into an external medium containing 0.2% polysorbate 80 PBS (pH 7.4) solution. The dialysis bags were then shaken at a speed of 100 rpm at 37 °C. Samples of 1 mL were taken from the dialysis buffer at different time intervals (0, 1, 2, 4, 8, 12, 24, 36, 48, 60, and 72 h), and 1 mL of fresh PBS containing 0.2% polysorbate 80 was replenished. The amounts of released PTX were determined by analytical reverse-phase high-performance liquid chromatography (RP-HPLC) (acetonitrile:water = 50:50 (v:v); flow rate: 1 mL/min; wavelength: 227 nm).

### 3.9. Cell Lines and Cell Culture

Human prostate cancer cells (DU145) were cultivated in DMEM supplemented with fetal bovine serum (FBS, 10%), streptomycin (100 μg/mL), and penicillin (100 units/mL). The cells were incubated in a humid atmosphere with 5% CO_2_ at 37 °C.

### 3.10. Cytotoxicity Assay

The in vitro cytotoxicities of PTX-loaded NPs were measured by MTT assays. Cells were seeded in 96-well plates (5000–8000 cells per well) and incubated at 37 °C. After 24 h, the medium was replaced with 180 μL of fresh medium, and the cells were treated with serial dilutions of PTX/PBA NPs, PTX/mPEG-PAL NPs, blank PBA NPs, and blank mPEG-PAL NPs (20 μL) for 48 h at 37 °C. At the end of the incubation, to each well was added 30 mL of MTT solution (5 mg/mL in PBS). After 4 h, the MTT solution was removed from the wells, followed by the addition of supplementary DMSO (100 μL) to dissolve the purple MTT formazan crystals. The absorbance of each well was determined at 490 nm on a microplate reader (Flexstation 3, Molecular Devices LLC, Sunnyvale, CA, USA).

### 3.11. Cellular Uptake

The intracellular uptake of NPs was analyzed by fluorescent microscopy. DU145 cells were seeded in 24-well plates (20,000 cells per well) and incubated at 37 °C for 12 h. Then, the cells were incubated with serial dilutions of coumarin-6 (C6) formulated NPs (C6/PBA NPs and C6/mPEG-PAL NPs, 50 µg/mL to 400 µg/mL). After incubation for 4 h, the medium was removed, and the cells were washed with PBS and fixed with 4% paraformaldehyde and DAPI staining for the observation with a fluorescence microscope (Axiotech Vario 3D, Carl Zeiss, Oberkochen, Germany).

### 3.12. Pharmacokinetics Studies

Pharmacokinetics was evaluated in ICR mice (about 20 g). The healthy ICR mice were randomly divided into three groups (n = 5 mice per group) and injected intravenously with Taxol, PTX/PBA NPs, and PTX/mPEG-PAL NPs. A total of 0.1 mL of fresh blood was collected through orbital at appropriate intervals, put into a centrifuge tube pretreated with heparin sodium, and centrifugated (5000 rpm, 10 min). A total of 100 µL acetonitrile was added to 50 µL of supernatant collected to extract PTX and precipitate protein in serum, and the mixture was centrifugated for another 10 min (1200 rpm). The amounts of PTX in the collected supernatant were determined by HPLC (acetonitrile:water = 50:50 (v:v); flow rate: 1.0 mL/min; wavelength: 227 nm).

### 3.13. In Vivo Antitumor Activity

BALB/c nude mice (4–5 weeks old) were used for the evaluation of the antitumor activities of the nanotherapies. The human prostate cancer cell line DU145 was grown to 80% confluence in 90 mm tissue culture dishes. After cell harvesting, the cells were resuspended in PBS at 4 °C to reach a final concentration of 1.5 × 10^7^ cells/mL. The right flanks of the BALB/c nude mice were subcutaneously injected with 200 μL of a cell suspension containing 3 × 10^6^ cells. At 4 days after implantation, the tumors reached approximately 150 mm^3^ in volume, and then the mice were randomly divided into four groups (n = 6 mice per group). Mice bearing DU145 tumor xenografts were injected intravenously with PBS, Taxol, PTX/PBA NPs, and PTX/mPEG-PAL NPs at 7.5 mg/kg three times on days 0, 3, and 6. Tumor volumes and body weights were monitored and recorded once every three days. The lengths (L) and widths (W) of the tumors were measured with calipers, and the tumor volume was calculated by the following formula: V = (L × W^2^)/2, where W is shorter than L.

### 3.14. Histologic Analysis

The tumor tissue and other organs from the sacrificed mice were excised at 15 d of the treatments with various drugs. After being fixed in n 4% formaldehyde, dehydrated with ethanol, and embedded in paraffin, the tumor tissue and organs were further sectioned into 5 μm slices for hematoxylin and eosin (H&E) staining and the terminal deoxynucleotidyl transferase-mediated dUTP nick-end labeling (TUNEL) staining assay. The H&E-stained tissues and TUNEL-stained tissues were imaged by fluorescence microscopy (NIKON ECLIPSE C1, Tokyo, Japan).

### 3.15. Statistical Analysis

Statistical analysis was performed using GraphPad Prism Version 8.0 software. Statistical difference was analyzed by *t*-test or two-way ANOVA. Two-tailed *p* values for each statistical test and sample size were indicated in the legends of the figures. All of the data are presented as the mean ± SD.

## 4. Conclusions

The development of highly pH-sensitive polymeric vehicles is of great importance to achieve targeting delivery of antitumor drugs as well as reducing the cytotoxicity of chemotherapies. In this study, we successfully constructed a pH-responsive nano-drug delivery system via dynamically conjugating hydrophobic polymers with pendent polyphenyl boronic acid groups (PBA-PAL) and dopamine-terminated hydrophilic polymers (mPEG-DA). Based on dynamic boronic acid/ester transformation, PTX-loaded nanoparticles with these two polymers were prepared by a nanoprecipitation method. The resulting PTX/PBA NPs showed appropriate particle sizes and PDIs and high drug encapsulation efficiency as well as sensitive pH-responsiveness. In addition, the in vitro drug release assays showed that PTX/PBA NPs had excellent pH-triggered drug-release capacity. Moreover, the in vitro and in vivo evaluations of antitumor activity showed that PTX/PBA NPs could effectively improve the pharmacokinetics of drugs and exhibited high therapeutic efficacy and low systemic toxicity. Boronic acid/ester-based pH-responsive nanodrugs prepared in this study can improve the therapeutic effect of anticancer drugs and is of high potential for clinical transformations.

## Figures and Tables

**Table 1:** Molecular weight and chemical composition of PBA-PAL and mPEG-DA copolymers.

Polymers	[AVL]:[LA] (Theoretical Molar Ratio)	[AVL]:[LA] (Actual Molar Ratio)	*Mn*, _NMR_*^a^* (g/mol)	*Mn*, _GPC_*^b^* (g/mol)	PDI *^c^*	Grafting Rates (%) *^d^*
PAL_2K_	1:1	1.1:1	2200	2300	1.32	-
PAL_4K_	1:1	1.1:1	4300	4200	1.37	-
PBA-PAL_2K_	1:1	1.1:1	3400	3700	1.38	85.1
PBA-PAL_4K_	1:1	1.1:1	6400	7200	1.39	80.2
mPEG_2K_-DA	-	-	2400	3100	1.04	
mPEG_4K_-DA	-	-	4300	4700	1.02	

*^a^*^,*d*^ Characterized by ^1^H NMR; *^b^*^,*c*^ Characterized by GPC.

**Table 2:** Characterization of PTX/PBA NPs prepared with PBA-PAL_2K_ and mPEG_2K_-DA.

PTX/PBA NPs	[PBA-PAL_2K_]:[mPEG_2K_-DA] Molar Ratio	Size (nm)	PDI	EE (%)	DL (%)
**1**	1:7	111.9 ± 1.64	0.122 ± 0.001	96.1	2.0
**2**	1:3.5	126.9 ± 2.56	0.143 ± 0.004	70.0	2.3
**3**	1:1	116.4 ± 4.21	0.133 ± 0.004	60.8	3.8
**4**	1:0.5	109.58 ± 2.89	0.174 ± 0.014	55.0	1.1

**Table 3:** Characterization of PTX/PBA NPs prepared with different PBA-PAL and mPEG-DA.

PTX/PBA NPs	*M_n,_* _PBA-PAL_-*M_n_*_, mPEG-DA_ (g/mol)	Size (nm)	PDI	EE (%)	DL (%)
**1**	2K−2K	111.9 ± 1.64	0.122 ± 0.001	96.1	2.0
**2**	2K−4K	136.9 ± 1.26	0.103 ± 0.001	49.7	0.6
**3**	4K−4K	133.5 ± 3.85	0.103 ± 0.001	43.1	0.5
**4**	4K−2K	79.88 ± 4.69	0.184 ± 0.017	23.7	0.5

**Table 4:** Pharmacokinetic parameters of PTX NPs and Taxol.

Pharmacokinetic Parameters	PTX/PBA NPs	PTX/mPEG-PAL NPs	Taxol
t1/2α (h)	0.195	0.153	0.054
t1/2β (h)	7.364	3.395	0.434
AUC (0–t) (μg/L·h)	270.465	114.049	67.456
AUC (0–∞) (μg/L·h)	589.846	118.627	85.372
Cmax (μg/L)	120.677	101.66	94.619

## Data Availability

The data presented in this study are available on request from the corresponding author.

## Supplementary Materials

The following supporting information can be downloaded at: https://www.mdpi.com/article/10.3390/molecules28114461/s1, Figure S1. ^1^H NMR spectra of AVL monomer. Figure S2. ^1^H NMR spectra of PAL_4K_. Figure S3. ^1^H NMR spectra of 4-bromomethyl-3-fluorophenylboronic acid. Figure S4. ^1^H NMR spectra of 4-azidomethyl-3-fluorophenylboronic acid. Figure S5. ^1^H NMR spectra of PBA-PAL_4K_. Figure S6. ^1^H NMR spectra of mPEG_4K_-DA. Figure S7. ^1^H NMR spectra of mPEG_2K_-OTS. Figure S8. ^1^H NMR spectra of mPEG_2K_-N_3_. Figure S9. ^1^H NMR spectra of mPEG-*g*-PAL 1. Figure S10. ^1^H NMR spectra of mPEG-*g*-PAL 2. Figure S11. ^1^H NMR spectra of mPEG-*g*-PAL 3. Table S1. Characterization of PTX/mPEG-PAL NPs.

## Sample Availability

Samples of the compounds are available from the authors.
