# Supplementary material for: Efficient pH-Responsive Nano-Drug Delivery System Based on Dynamic Boronic Acid/Ester Transformation
Source: Molecules. 2023 May 31;28(11):4461. doi: 10.3390/molecules28114461 (PMC10254657; doi:10.3390/molecules28114461)
Supplement: Supplementary file 1 [file molecules-28-04461-s001.zip › molecules-2361303-supplementary.pdf]

Supporting Information

Efficient pH-Responsive Nano-Drug Delivery System Based on Dynamic Boronic Acid/Ester Transformation

*Weijun Chen[1], Wanxuan Xie[2], Guangkuo Zhao[2] and Qi Shuai[2,]**

[1] Department of Child Health Care, Children's Hospital, Zhejiang University School of Medicine, National Clinical Research Center for Child Health, Hangzhou, Zhejiang 310052, P, R. China
[2] Collaborative Innovation Center of Yangtze River Delta Region Green Pharmaceuticals, Zhejiang University of Technology, Hangzhou, Zhejiang 310014, P. R. China

* Corresponding author
Qi Shuai - Collaborative Innovation Center of Yangtze River Delta Region Green Pharmaceuticals, Zhejiang University of Technology, Hangzhou, Zhejiang 310014, P. R. China; E-mail: qshuai@zjut.edu.cn

Table of Contents

1. Experimental Section

2. Supporting Data

Figure S1. ^{1}H NMR spectra of AVL monomer.

Figure S2. ^{1}H NMR spectra of PAL_{4K}.

Figure S3. ^{1}H NMR spectra of 4-bromomethyl-3-fluorophenylboronic acid.

Figure S4. ^{1}H NMR spectra of 4-azidomethyl-3-fluorophenylboronic acid.

Figure S5. ^{1}H NMR spectra of PBA-PAL_{4K}.

Figure S6. ^{1}H NMR spectra of $mPEG_{4K}$-DA.

Figure S7. ^{1}H NMR spectra of $mPEG_{2K}$-OTS.

Figure S8. ^{1}H NMR spectra of $mPEG_{2K}$-N_3.

Figure S9. ^{1}H NMR spectra of mPEG-*g*-PAL **1**.

Figure S10. ^{1}H NMR spectra of mPEG-*g*-PAL **2**.

Figure S11. ^{1}H NMR spectra of mPEG-*g*-PAL **3**.

Table S1. Characterization of PTX/mPEG-PAL NPs.

1. Experimental Section

Synthesis of α-propargyl-δ-valerolactone (AVL).

Lithium diisopropylamide (LDA, 55.1 mL, 110.0 mmol) was added dropwise to anhydrous tetrahydrofuran (THF, 300 mL), followed by dropwise addition of δ-valolactone (250 mL, 0.4 M in THF) at -78 °C. The mixture was then stirred for 40 min. Next, a mixture of bromoproparynes (13.4 mL, 120.0 mmol) and hexamethylphosphoramide (20.9 mL, 120.0 mmol) was added dropwise, and stirring continued for 2 hours at -30 °C. The reaction was quenched by excessive saturated ammonium chloride solution and the mixture was stirred for another 30 min. THF was removed by rotary evaporator and the residue was extracted with ether (3 × 100 mL). The combined organic layer was washed with saturated sodium chloride solution, dried over anhydrous Na_2SO_4, filtered, and concentrated under vacuum. The crude residue was purified by flash column chromatography on silica gel (hexane: EA = 9:1-6:1) to give AVL (6.15 g, 45%). AVL for polymerization was further purified by vacuum distillation. **^{1}H NMR** (400 MHz, CDCl3, δ): 4.44–4.23 (m, 2H), 2.78–2.60 (m, 2H), 2.59–2.44 (m, 1H), 2.30 (d, *J* = 13.5, 7.0 Hz, 1H), 2.03 (t, *J* = 2.6 Hz, 1H), 1.99–1.89 (m, 2H), 1.81–1.66 (m, 1H).

Synthesis of 4-azidomethyl-3-fluorophenylboronic acid.

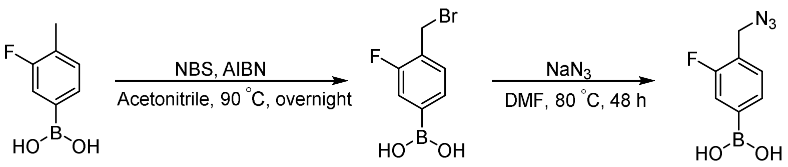

4-bromomethyl-3-fluorophenylboronic acid: 3-fluoro-4-methylphenylboronic acid (1.5 g, 9.7 mmol) was dissolved in acetonitrile (100 mL), followed by addition of N-bromosuccinimide (NBS, 2.08 g, 11.7 mmol) and azodiisobutyronitrile (AIBN, 16.4 mg, 0.1 mmol). The mixture was stirred at 90 °C for 4 h. After reaction, organic solvent was removed by rotary evaporator and the residue was dissolved in EA (100 mL) and washed with water.

The aqueous phase was extracted twice with EA (2 × 50 mL). The combined organic layer was then washed with brine, dried over anhydrous Na_2SO_4, filtered, and concentrated under vacuum. The residue was purified by flash column chromatography on silica gel (DCM: MeOH = 200: 1) to afford 4-bromomethyl-3-fluorophenylboronic acid (1.3 g, 58%). **^{1}H NMR** (400 MHz, DMSO-d_6, δ): 8.29 (s, 2H), 7.60-7.44 (m, 3H), 4.63 (d, 2H).

4-azidomethyl-3-fluorophenylboronic acid: 4-bromomethyl-3-fluorophenylboronic acid (1.3 g, 5.6 mmol) and sodium azide (1.1 g, 16.7 mmol) were dissolved in DMF (25 mL) and then stirred at 80 °C for 48 h. After reaction, organic solvent was removed by rotary evaporator and the residue was dissolved in EA (100 mL) and washed with water. The aqueous phase was extracted twice with EA (2 × 50 mL). The combined organic layer was dried over anhydrous Na_2SO_4, filtered, and concentrated under vacuum to give 4-azidomethyl-3-fluorophenylboronic acid (0.92 g, 85%). **^{1}H NMR** (400 MHz, DMSO-d_6, δ): 8.27 (s, 2H), 7.72-7.37 (m, 3H), 4.50 (d, 2H).

Synthesis of mPEG$_{2K}$-N$_3$.

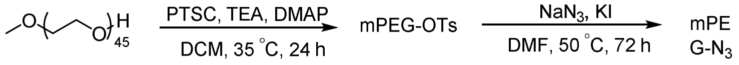

mPEG$_{2K}$-OTs: A solution of mPEG$_{2K}$ (5 g, 2.5 mmol), 4-dimethylaminopyridine (30.54 mg, 0.25 mmol) and triethylamine (1.74 mL, 12.5 mmol) in anhydrous DCM (50 mL) was stirred at 0 °C for 5 min. Then, a solution of *p*-toluenesulfonyl chloride (PTSC, 4.43 g, 75 mmol) in 2 mL DCM was added dropwise into the mixture, followed by stirring at 35 °C for another 24 h. After reaction, the mixture was diluted with DCM (100 mL) and the mixture was washed with 1 M HCl. The combined organic layer was dried over anhydrous Na_2SO_4, filtered, and concentrated under vacuum. The residue was purified by precipitation from DCM: diethyl ether (1:10) for three times to afford the mPEG$_{2K}$-OTs (4.5 g, 82.3%). **^{1}H NMR** (400 MHz, $CDCl_3$, δ): 7.80 (d, 2H), 7.34 (d, 2H), 4.15 (m, 2H), 3.85–3.42 (m, 181H), 3.37 (s, 3H), 2.15 (s, 3H).

$mPEG_{2K}$-N_3: A solution of $mPEG_{2K}$-OTs (4.5 g, 2.0 mmol) and potassium iodide (34.9 mg, 0.2 mmol) in acetone (80 mL) was stirred at room temperature for 5 min. Then, a solution of sodium azide (301 mg, 4.6 mmol) in 6 mL water was added dropwise into the mixture, followed by stirring at at 50 °C for another 48 h. After reaction, acetone was removed by rotary evaporator and the residue was extracted with DCM twice (2 × 100 mL). Then the combined organic layer was dried over anhydrous Na_2SO_4, filtered, and concentrated under vacuum. The residue was purified by precipitation from DCM: hexane (1: 10) for three times to afford the $mPEG_{2K}$-N_3 (3.66 g, 84.5%). **^{1}H NMR** (400 MHz, $CDCl_3$, δ): 3.85-3.42 (m, 181H), 3.37 (s, 3H).

General Procedures for the Synthesis of mPEG-*g*-PAL copolymer.

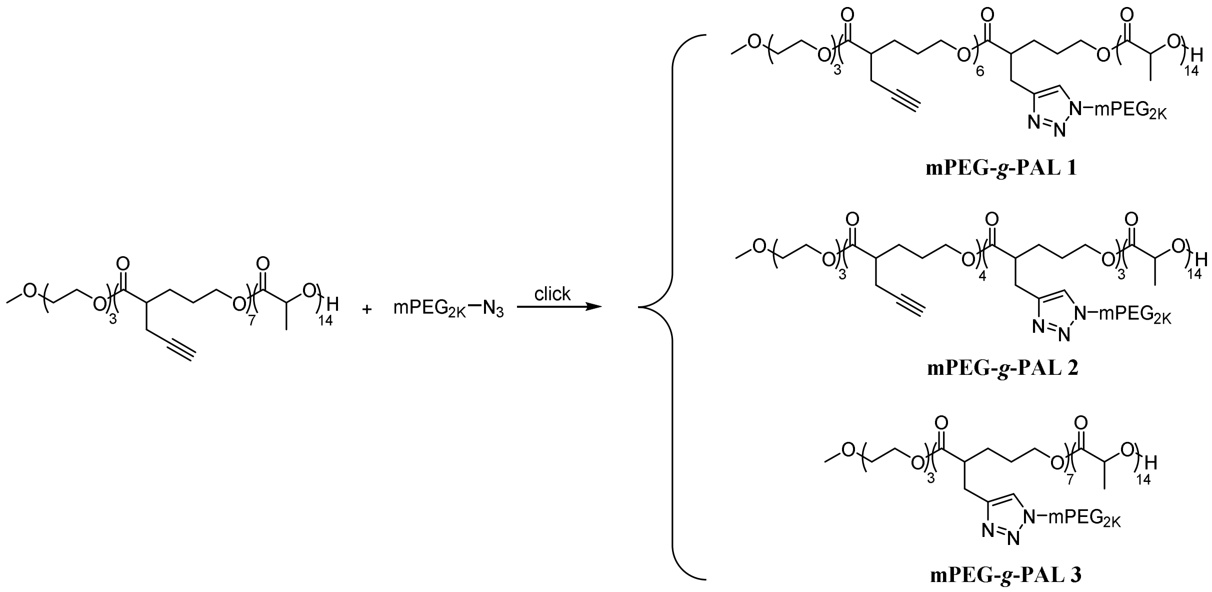

PAL_{2K} (1 equiv.), $mPEG_{2K}$-N_3 (1, 3, or 7 equiv.), cuprous bromide (1 equiv.), and pentaethylenetriamine (1 eq) were added to anhydrous DMF under N_2. The mixtuer was stirred in dark at 50 °C for 24 h. After reaction, the mixture was dialyzed in water for 2 d (molecular weight cutoff of 4 kD) and vacuum freeze-drying afforded mPEG-*g*-PAL copolymers (71%-86%).

*mPEG-g-PAL **1*** (1 equiv. of $mPEG_{2K}$-N_3): **^{1}H NMR** (400 MHz, $CDCl_3$, δ): 7.52 (s, 1H), 5.38-4.95 (m, 14H), 4.55-4.04 (m, 17H), 3.98-3.43 (m, 181H), 3.64 (m, 6H), 3.17-2.30 (m, 21H), 2.00 (s, 6H), 1.89-1.23 (m, 70H).

mPEG-g-PAL ***2*** (3 equiv. of $mPEG_{2K}$-N_3): **^{1}H NMR** (400 MHz, $CDCl_3$, δ): 7.52 (s, 3H), 5.38-4.95 (m, 14H), 4.55-4.04 (m, 17H), 3.98-3.43 (m, 543H), 3.64 (m, 12H), 3.17-2.30 (m, 21H), 2.00 (s, 4H), 1.89-1.23 (m, 70H).

mPEG-g-PAL ***3*** (7 equiv. of $mPEG_{2K}$-N_3): **^{1}H NMR** (400 MHz, $CDCl_3$, δ): 7.52 (s, 7H), 5.38-4.95 (m, 14H), 4.55-4.04 (m, 17H), 3.98-3.43 (m, 1267H), 3.64 (m, 24H), 3.17-2.30 (m, 21H), 1.89-1.23 (m, 70H).

2. Supporting Data

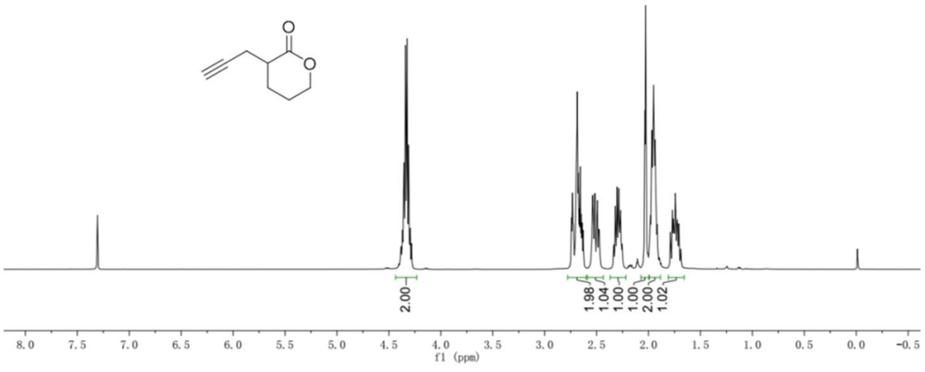

Figure S1. ^{1}H NMR spectra of AVL monomer.

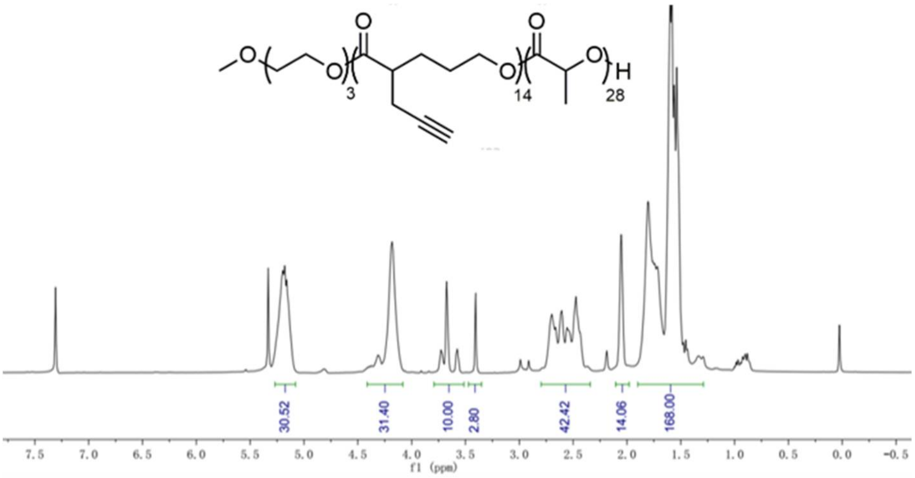

Figure S2. ^{1}H NMR spectra of PAL_{4K}.

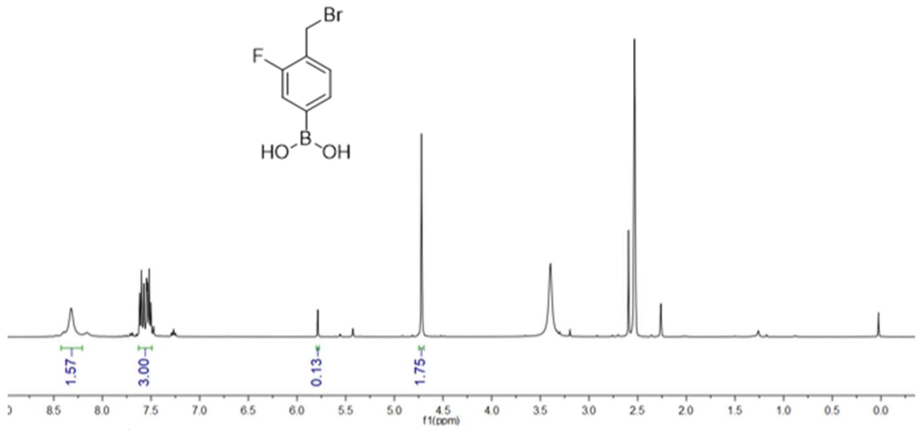

Figure S3. ^{1}H NMR spectra of 4-bromomethyl-3-fluorophenylboronic acid.

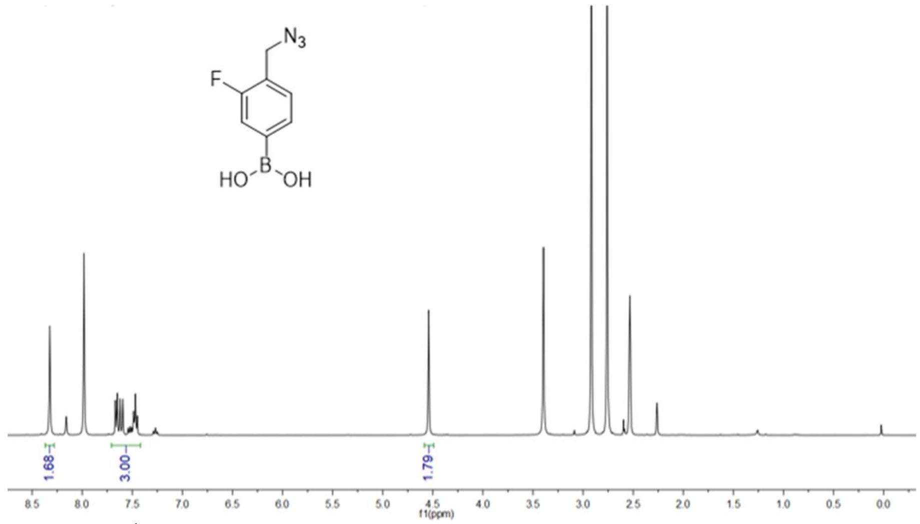

Figure S4. ^{1}H NMR spectra of 4-azidomethyl-3-fluorophenylboronic acid.

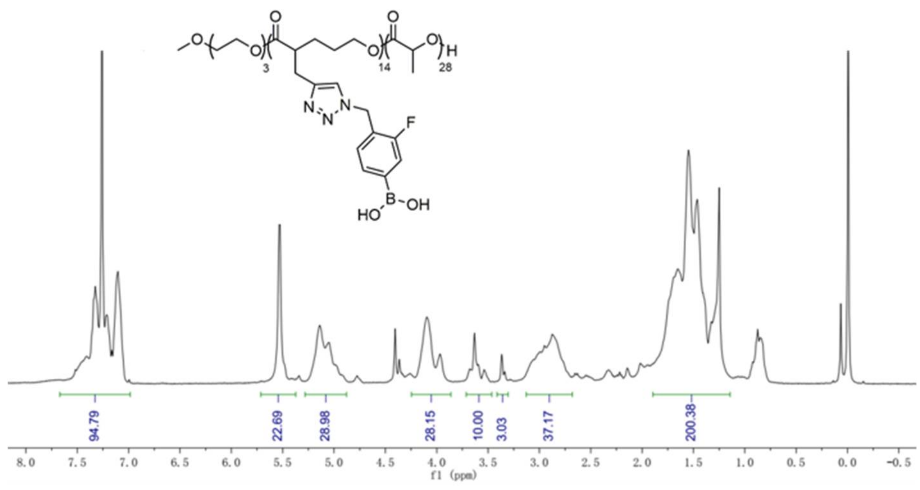

Figure S5. ^{1}H NMR spectra of PBA-PAL$_{4K}$.

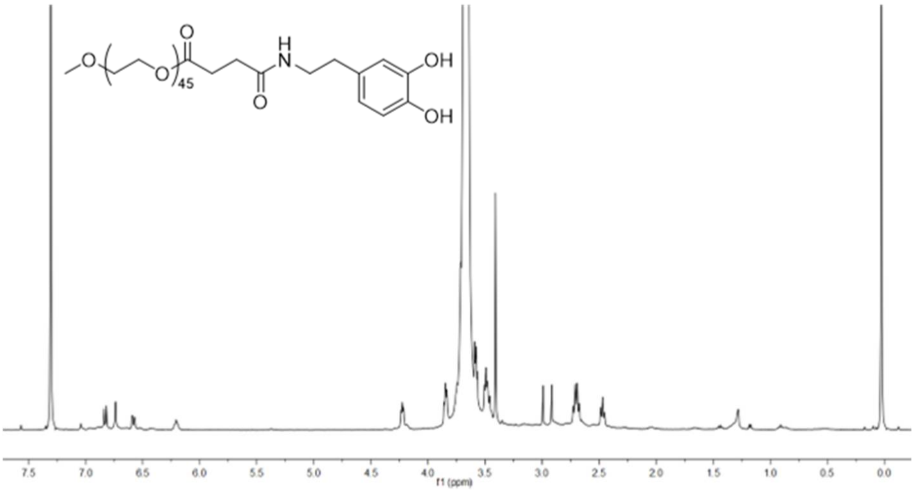

Figure S6. ^{1}H NMR spectra of mPEG$_{4K}$-DA.

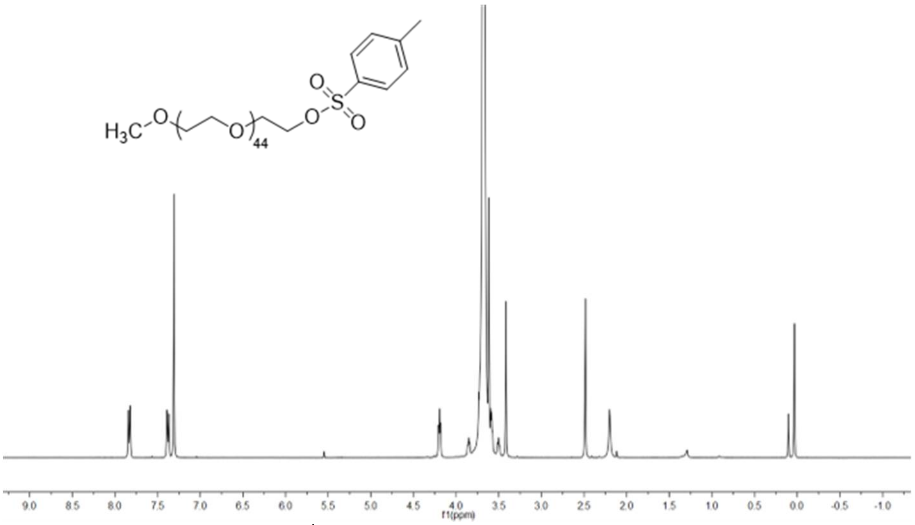

Figure S7. ^{1}H NMR spectra of mPEG$_{2K}$-OTS.

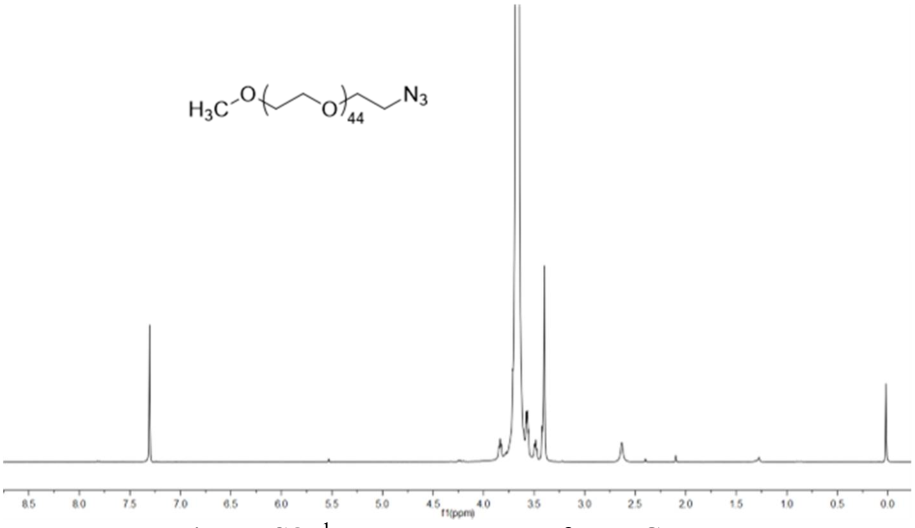

Figure S8. ^{1}H NMR spectra of mPEG$_{2K}$-N_3.

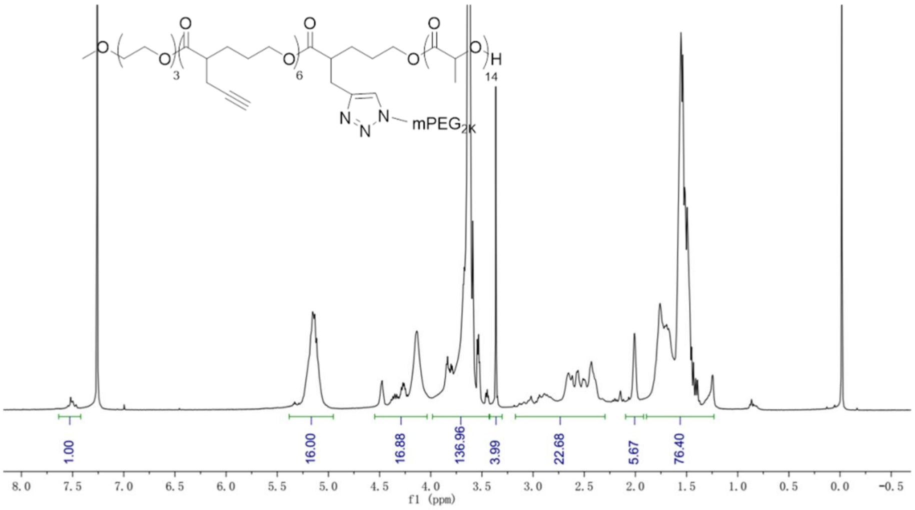

Figure S9. ^{1}H NMR spectra of mPEG-*g*-PAL **1**.

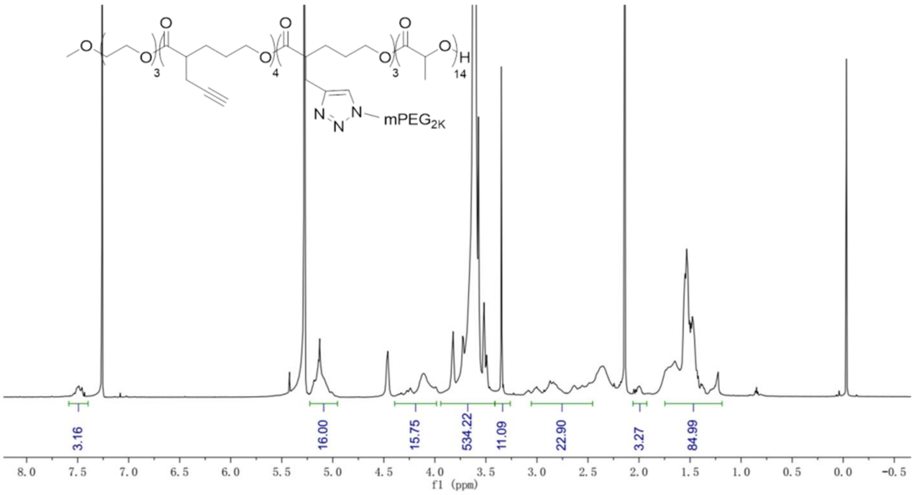

Figure S10. ^{1}H NMR spectra of mPEG-*g*-PAL **2**.

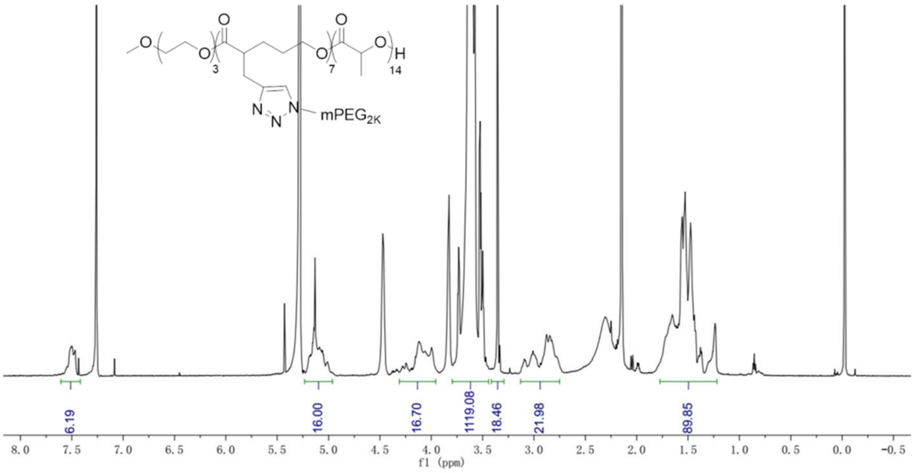

Figure S11. ^{1}H NMR spectra of mPEG-*g*-PAL **3**.

Table S1. Characterization of PTX/mPEG-PAL NPs.

PTX/mPEG-PAL NPs	Polymer	Size (nm)	PDI	EE (%)	DL (%)
**1**	mPEG-*g*-PAL **1**	54.08 ± 2.59	0.185 ± 0.005	80.2	3.1
**2**	mPEG-*g*-PAL **2**	60.13 ± 4.67	0.443 ± 0.221	25.1	1.2
**3**	mPEG-*g*-PAL **3**	n.a.	n.a.	n.a.	n.a.

n.a.: not available.
